# U-shaped relationship found between fibrinogen-to-albumin ratio and systemic inflammation response index in osteoporotic fracture patients

**DOI:** 10.1038/s41598-024-61965-9

**Published:** 2024-05-17

**Authors:** Xiao-jie Zhou, Ke Lu, Zhou-hang Liu, Min-zhe Xu, Chong Li

**Affiliations:** Department of Orthopedics, Affiliated Kunshan Hospital of Jiangsu University, No. 566 East of Qianjin Road, Suzhou, 215300 Jiangsu China

**Keywords:** Fibrinogen‑to‑albumin ratio, Systemic inflammation response index, Osteoporosis, Inflammation, Medical research, Risk factors

## Abstract

The relationship between the Systemic Inflammatory Response Index (SIRI) and the Fibrinogen-to-albumin ratio (FAR) has not been extensively investigated. The objective of this study was to determine the independent relationship between FAR and SIRI in people with osteoporotic fractures (OPF). A cross-sectional study was conducted using retrospective data from 3431 hospitalized OPF patients. The exposure variable in this study was the baseline FAR, while the outcome variable was the SIRI. Covariates, including age, gender, BMI, and other clinical and laboratory factors, were adjusted. Cross-correlation analysis and linear regression models were applied. The generalized additive model (GAM) investigated non-linear relationships. Adjusted analysis revealed an independent negative association between FAR and SIRI in OPF patients (β = − 0.114, p = 0.00064, 95% CI − 0.180, − 0.049). A substantial U-shaped association between FAR and SIRI was shown using GAM analysis (p < 0.001). FAR and SIRI indicated a negative association for FAR below 6.344% and a positive correlation for FAR over 6.344%. The results of our study revealed a U-shaped relationship between SIRI and FAR. The lowest conceivable FAR for a bone-loose inflammatory disease might be 6.344%, suggesting that this has particular significance for the medical diagnosis and therapy of persons with OPF. Consequently, the term "inflammatory trough" is proposed. These results offer fresh perspectives on controlling inflammation in individuals with OPF and preventing inflammatory osteoporosis.

## Introduction

Osteoporosis (OP) is a common bone condition that significantly affects the health and quality of life of individuals worldwide^1^. On a global scale, OP impacts around 200 million individuals^2^. According to a 2019 study, the prevalence of OP in Chinese women and men over 50 years was as high as 29.13% and 6.46%, respectively^3^. Research on reducing the prevalence and determining the causes of OP has become a crucial focus in public health. Researchers have recently shifted their focus towards the crucial role that inflammation plays in the development and origin of OP. There is a strong connection between OP risk and inflammation. Chronic inflammation can potentially raise the risk of both OP and fractures^4^.

Two recently proposed measures of systemic inflammation are the Fibrinogen-to-albumin ratio (FAR) and the Systemic Inflammatory Response Index (SIRI)^5,6^. The FAR assesses coagulation and inflammatory status by evaluating the fibrinogen-to-albumin ratio, whereas SIRI evaluates systemic inflammation by considering the ratio of platelets, neutrophils, and lymphocytes^7,8^. When assessing systemic inflammation and forecasting the prognosis of a disease, the FAR and SIRI are highly relevant and useful^9,10^. They provide physicians with essential information on the inflammatory status and prognosis of their patients, which aids in the decision-making process about treatment and disease progression. When compared to recognized inflammatory markers, the FAR and SIRI exhibit more therapeutic potential and predictive value^11^. They provide a more thorough representation of systemic inflammation and more precise recommendations for illness prevention, diagnosis, and therapy at an early stage.

These two are widely used inflammatory markers that have been widely applied in determining inflammatory status and predicting illness outcomes^5,6^. Nevertheless, there is still additional knowledge to be gained regarding the potential correlation between baseline FAR and SIRI in individuals who have experienced osteoporotic fractures (OPF). This study aims to examine the existing correlation between the indicators of SIRI and FAR to enhance our understanding of their clinical significance and potential applications. It is worth noting that there is a lack of research on the evaluation of these indicators and their predictive capabilities for prognosis.

## Materials and methods

### Study design and participants

**Figure 1:**
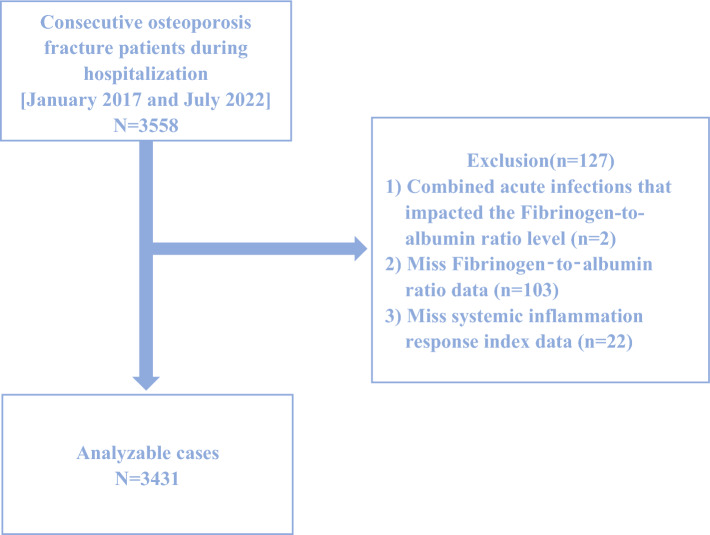
Study flow chart.

The patient data included in this retrospective study was collected from January 2015 to March 2022. The medical records of patients were acquired from Kunshan Hospital, an institution associated with Jiangsu University in Suzhou, China. The study comprised 3431 OPF patients in total. During their hospital stay, medical blood tests were performed on each of them. OPFs, sometimes referred to as fragility fractures, are low-energy fractures that happen when someone falls from a height of standing or less. They have the potential to significantly raise the risk of further fragility fractures^12^. The presence of OP and the concurrent absence of other metabolic bone disorders are prerequisites for the diagnosis of OPFs. The following were the inclusion criteria for OP: (1) the diagnosis of OP based on a T-Score of − 2.5 or lower, even in the absence of significant bone fractures; and (2) the incidence of bone instability and fractures without other metabolic bone illnesses, accompanied by physiological bone density (T-Score)^13^. The following patients have been eliminated from consideration: (1) those who had acute infections that impacted the Fibrinogen-to-albumin ratio level (n = 2); (2) those who had missing FAR data (n = 103); and (3) those who had missing SIRI data (n = 22). A total of 3431 patients all met the study's inclusion criteria after the application of the criteria. Figure 1 shows a schematic diagram of the patient selection procedure. The study adheres to the Helsinki Declaration and received approval from the Kunshan Hospital Ethics Committee at Jiangsu University (Approval No. 2021-06-016-K01). The patients' identities were concealed to facilitate an impartial investigation. Each patient provided written informed consent.

### Outcome variables

The measurements were obtained from the initial fasting blood sample collected within 24 h of admission, using the same device and routine operating procedure, by trained operators. The formula used to calculate SIRI, which was the dependent variable in our investigation, was SIRI = neutrophil count × monocyte count ÷ lymphocyte count^14^. The Sysmex XN-10 (B4) hematology analyzer was used to measure the number of neutrophils, monocytes, and lymphocytes using nuclear staining and flow cytometry assays.

### Exposure variables

In this investigation, the exposure variable was FAR, which was computed as follows: FAR (%) = FIB (g/L)/ALB (g/L) × 100%^15^. The calculation of the FAR involved dividing the concentrations of fibrinogen and albumin for each patient. The fibrinogen concentrations were quantified through coagulation analysis utilizing the CN-6000 automated coagulation analyzer, while the albumin concentrations were assessed using the Beckman AU5800 biochemical analyzer pipeline using biuret analysis.

### Covariates

The variables in the study included age, gender, body mass index (BMI), American Society of Anesthesiologists (ASA) score, calcium, urea nitrogen (UN), creatinine (Cr), uric acid (UA), aspartate aminotransferase (AST), and parathyroid hormone (PTH). Upon admission to the hospital, all clinical variables were measured within 3 days.

### Statistics

For continuous and categorical variables, the results are displayed as means ± standard deviations (SDs), median (Q1, Q3), and frequency (%), accordingly. For univariate data measured in absolute values, either Fisher's exact test or Pearson's chi-squared test were utilized. The t-test was employed for continuous variables in standard data, whereas the Mann–Whitney *U* test was utilized for non-normally distributed continuous data. A univariate linear regression analysis was conducted to evaluate the correlation between the FAR and SIRI in patients with OPF.

The study analyzed patients with OPF to determine if there was a direct relationship between FAR and SIRI. This was done using the generalized estimation equation (GEE) with proper adjustment for covariables. The models that were generated were completely calibrated (model 3/4), partially calibrated (model 2), and not calibrated (model 1). The following criteria were used to determine how these covariates should be adjusted when the collinearity of the covariances was first detected using variance infection factor analysis: Covariates that meet criteria 1 or indicated covariates of P < 0.1 in the univariate model; (1) a change of matching odds ratio (OR) of ≥ 10% was detected when covariates were added to the basic model or removed from the entire model^16^. Standard 1 and Standard 2 were used to alter Model 3 and Model 4, respectively. Finally, four models were determined: model 1 was not calibrated; Model 2 (partially calibrated) was modified based on factors, such as age, gender, BMI, ASA level; Model 3 is adjusted according to age, gender, BMI, ASA level and UA, Cr, UN, AST; Model 4 was modified based on factors, such as age, gender, BMI, ASA, UA, Cr, UN, AST, PTH, and calcium.

The detection of potential non-linear relationships was performed using a generalized additive model (GAM). A two-piecewise linear regression model was used to identify threshold effects in the smoothing curves when there were significant relationships. An algorithmic approach utilizing a maximum likelihood model was used to recursively calculate the inflection point for these unique ratio curves^17^. After classifying patients according to specific covariates, we conducted additional studies to evaluate the reliability of the findings and to compare the variations across different groups of patients. The examination of subgroup interactions and modifications was conducted using the likelihood ratio test (LRT).

Empower Stats (www.empowerstats.com, X&Y Solutions, Inc., Boston, MA, USA) was used for all statistical analyses. Additionally, R 3.6.3 (http://www.r-project.org) was utilized. *P*-values were considered statistically significant if they were less than 0.05.

### Ethics approval and consent to participate

We received ethical approval from the Affiliated Kunshan Hospital of Jiangsu University (approval No. 2021-06-015-K01), and was compliant with the Declaration of Helsinki. Patient identification data were hidden from the researchers analyzing the data. The patients signed informed consent.

## Results

### Patient characteristics

Table 1 summarizes the baseline characteristics of 3431 patients with OPF who were admitted between January 2015 and March 2022 and were placed within the specified FAR quartiles. The mean age of these patients (31.97% male, 68.03% female) was 69.62 ± 11.80 years. The average FAR was 4.54 ± 1.01, and the average SIRI was 3.44 ± 3.74. Based on FAR (< 5.5875%, 5.5875–6.8018%, 6.8018–8.4130%, 6.4130–19.4871%), patients were grouped into quartiles. SIRI, UA, Cr, UN, AST, PTH, calcium, FEE, MAP, BTX, P1NP, and LDL showed significant differences.

**Table 1:** Patient characteristics based on FAR quartiles.

Characteristics	Total	Mean + SD/N(%)				*P*-value	*P*-value*
		Q1 (< 5.5875%)	Q2 (5.5875–6.8018%)	Q3 (6.8018–8.4130%)	Q4 (6.4130–19.4871%)		
N	3431	839	865	863	864		
SIRI	3.4364 ± 3.7410	4.4919 ± 4.9060	3.2797 ± 3.5036	2.8290 ± 2.8987	3.1749 ± 3.1663	< 0.001	< 0.001
UA, μmol/L	285.0143 ± 92.3169	305.1740 ± 90.5832	285.2451 ± 85.2208	276.9768 ± 91.1922	273.2350 ± 98.6341	< 0.001	< 0.001
Cr, μmol/L	66.3594 ± 40.0476	64.1704 ± 17.2010	63.7861 ± 19.4147	65.4647 ± 27.7304	71.9549 ± 69.9869	< 0.001	0.330
UN, μmol/L	6.0765 ± 3.3650	5.6023 ± 1.8270	6.0443 ± 4.9251	5.9956 ± 2.2807	6.6501 ± 3.4243	< 0.001	< 0.001
AST, U/L	26.4252 ± 21.8906	30.4839 ± 35.0601	25.6289 ± 11.8828	24.4658 ± 11.5722	25.2384 ± 20.3459	< 0.001	< 0.001
PTH, pmol/L	14.7038 ± 10.4422	15.2561 ± 10.6049	14.7712 ± 10.6805	13.6952 ± 7.7403	15.2025 ± 12.2680	0.044	0.018
Calcium, mmol/L	2.2070 ± 0.1292	2.2148 ± 0.1398	2.2096 ± 0.1262	2.2042 ± 0.1241	2.1996 ± 0.1260	0.086	0.016
Fee, RMB	45,948.2536 ± 21,221.0929	45,646.1542 ± 18,269.0934	45,916.1792 ± 20,138.4311	46,963.1049 ± 26,751.4323	45,260.0466 ± 18,529.3744	0.383	0.812
SRBC, U	0.2932 ± 1.4078	0.3337 ± 1.4197	0.2214 ± 1.2806	0.3818 ± 1.8117	0.2373 ± 0.9909	0.052	0.040
BTX, μg/L	0.5304 ± 0.2789	0.4626 ± 0.2196	0.5006 ± 0.2901	0.5563 ± 0.2829	0.5649 ± 0.2862	0.002	0.001
P1NP, μg/L	58.1531 ± 35.8932	53.0339 ± 22.1772	60.2652 ± 52.2383	61.8284 ± 32.9173	55.9064 ± 27.1869	0.107	0.045
HDL, mmol/L	1.3420 ± 0.3091	1.3349 ± 0.3165	1.3391 ± 0.2859	1.3561 ± 0.2990	1.3362 ± 0.3328	0.633	0.506
LDL, mmol/L	2.5408 ± 0.7660	2.4554 ± 0.7277	2.4918 ± 0.7491	2.6430 ± 0.7858	2.5536 ± 0.7814	< 0.001	< 0.001
Gender, N (%)						0.973	–
Female	2334 (68.0268%)	574 (68.4148%)	585 (67.6301%)	584 (67.6709%)	591 (68.4028%)		
Male	1097 (31.9732%)	265 (31.5852%)	280 (32.3699%)	279 (32.3291%)	273 (31.5972%)		
Age 5 quantiles, y						0.375	–
Q0 (< 58 years)	633 (18.4494%)	141 (16.8057%)	170 (19.6532%)	168 (19.4670%)	154 (17.8241%)		
Q1 (58–66 years)	726 (21.1600%)	163 (19.4279%)	199 (23.0058%)	187 (21.6686%)	177 (20.4861%)		
Q2 (66–72 years)	678 (19.7610%)	179 (21.3349%)	173 (20.0000%)	163 (18.8876%)	163 (18.8657%)		
Q3 (72–80 years)	648 (18.8866%)	173 (20.6198%)	150 (17.3410%)	162 (18.7717%)	163 (18.8657%)		
Q4 (80–87 years)	746 (21.7429%)	183 (21.8117%)	173 (20.0000%)	183 (21.2051%)	207 (23.9583%)		
BMI categorical, N(%)						0.875	–
< 24 kg/m^2^	2114 (61.6147%)	515 (61.3826%)	533 (61.6185%)	528 (61.1819%)	538 (62.2685%)		
24–28 kg/m^2^	1079 (31.4486%)	265 (31.5852%)	267 (30.8671%)	283 (32.7926%)	264 (30.5556%)		
≥ 28 kg/m^2^	238 (6.9368%)	59 (7.0322%)	65 (7.5145%)	52 (6.0255%)	62 (7.1759%)		
ASA						0.228	–
1	311 (9.0644%)	89 (10.6079%)	84 (9.7110%)	75 (8.6906%)	63 (7.2917%)		
2	2308 (67.2690%)	547 (65.1967%)	590 (68.2081%)	592 (68.5979%)	579 (67.0139%)		
3	801 (23.3460%)	200 (23.8379%)	187 (21.6185%)	193 (22.3638%)	221 (25.5787%)		
4	11 (0.3206%)	3 (0.3576%)	4 (0.4624%)	3 (0.3476%)	1 (0.1157%)		

*FAR* Fibrinogen-to-albumin ratio, *SD* standard deviation, *Q1* first quartile, *Q2* second quartile, *Q3* third quartile, *Q4* fourth quartile, *SIRI* systemic inflammation response index, *UA* uric acid, *CR* creatinine, *UN* urea nitrogen, *AST* aspartate aminotransferase, *PTH* parathyroid hormone, *SRBC* suspended red blood cell, *BTX* botulinum toxin, *P1NP* N-terminal propeptide of type I procollagen, *HDL* high-density lipoproteins, *LDL* low-density lipoproteins, *BMI* body mass index, *ASA* American Society of Anesthesiologists.

*P*-value*: Kruskal Wallis Rank Test for continuous variables, Fisher Exact for categorical variables with Expects < 10.

### Univariate analyses of SIRI-related factors

Significant correlations between SIRI and factors, such as gender, UA, Cr, UN, AST, PTH, calcium, BTX, P1NP, LDL, and FAR were found in the univariate analysis (Table 2).

**Table 2:** Univariate analyses of factors associated with SIRI.

Characteristics	Statistics	β^a^ (95% CI) *P*-value
Gender, N(%)		
Female	2334 (68.027%)	Reference
Male	1097 (31.973%)	0.471 (0.203, 0.739) 0.001
Age 5 quantiles, years		
Q0 (< 58 years)	633 (18.449%)	Reference
Q1 (58–66 years)	726 (21.160%)	− 0.243 (− 0.641, 0.156) 0.233
Q2 (66–72 years)	678 (19.761%)	− 0.060 (− 0.466, 0.345) 0.770
Q3 (72–80 years)	648 (18.887%)	− 0.119 (− 0.529, 0.291) 0.570
Q4 (80–87 years)	746 (21.743%)	0.082 (− 0.314, 0.479) 0.684
BMI categorical, N(%)		
< 24 kg/m^2^	2114 (61.615%)	Reference
24–28 kg/m^2^	1079 (31.449%)	0.031 (− 0.243, 0.305) 0.824
≥ 28 kg/m^2^	238 (6.937%)	− 0.485 (− 0.986, 0.017) 0.058
ASA		
1	311 (9.064%)	Reference
2	2308 (67.269%)	− 0.178 (− 0.621, 0.265) 0.431
3	801 (23.346%)	− 0.196 (− 0.686, 0.294) 0.434
4	11 (0.321%)	0.644 (− 1.606, 2.894) 0.575
UA, μmol/L	285.014 ± 92.317	0.005 (0.004, 0.007) < 0.001
Cr, μmol/L	66.359 ± 40.048	0.006 (0.003, 0.009) 0.001
UN, mmol/L	6.076 ± 3.365	0.098 (0.061, 0.135) < 0.001
AST, U/L	26.425 ± 21.891	0.033 (0.027, 0.038) < 0.001
PTH, pmol/L	14.704 ± 10.442	0.028 (0.013, 0.043) 0.001
Calcium, mmol/L	2.207 ± 0.129	− 2.804 (− 3.769, − 1.838) < 0.001
Fee, RMB	45,948.254 ± 21,221.093	0.000 (− 0.000, 0.000) 0.202
SRBC, U	0.293 ± 1.408	0.079 (− 0.010, 0.167) 0.084
BTX, μg/L	0.530 ± 0.279	− 1.848 (− 2.628, − 1.067) < 0.001
P1NP, μg/L	58.153 ± 35.893	− 0.011 (− 0.017, − 0.005) 0.001
HDL, mmol/L	1.342 ± 0.309	0.289 (− 0.226, 0.803) 0.272
LDL, mmol/L	2.541 ± 0.766	− 0.692 (− 0.898, − 0.486) < 0.001
FAR, %	7.256 ± 2.413	− 0.141 (− 0.193, − 0.089) < 0.001
FAR quartile, %		
Q1 (< 5.5875%)	839 (24.454%)	Reference
Q2 (6.8018–8.4130%)	865 (25.211%)	− 1.212 (− 1.563, − 0.862) < 0.001
Q3 (6.8018–8.4130%)	863 (25.153%)	− 1.663 (− 2.014, − 1.312) < 0.001
Q4 (6.4130–19.4871%)	864 (25.182%)	− 1.317 (− 1.668, − 0.966) < 0.001

^a^Dependent variable SIRI, as a result of univariate analyses for SIRI.

*SIRI* systemic inflammation response index, *BMI* body mass index, *ASA* American Society of Anesthesiologists, *UA* uric acid, *Cr* creatinine, *UN* urea nitrogen, *AST* aspartate aminotransferase, *PTH* parathyroid hormone, *SRBC* suspended red blood cell, *BTX* botulinum toxin, *P1NP* N-terminal propeptide of type I procollagen, *HDL* high-density lipoproteins, *LDL* low-density lipoproteins, *FAR* Fibrinogen-to-albumin ratio.

### Examining the relationship between SIRI and FAR levels

Subsequently, the correlation between FAR and SIRI in patients with OPF was examined using four different models (Table 3). A noteworthy correlation between these factors was noted in unadjusted Model 1 (β = − 0.141, 95% CI − 0.193, − 0.089, P < 0.000001). Adjusted Model 2, which included variables including age, gender, body mass index (BMI), and ASA, showed a similar association (β = − 0.140, 95% CI − 0.191, − 0.088, P < 0.000001). A significant negative connection was also shown by controlled Model 3, which further controlled for UA, CR, UN, and AST (β = − 0.125, 95% CI − 0.176, − 0.073, P < 0.000001). A similar relationship between these parameters was identified in Adjusted Model 4, which also included adjustments for PTH and calcium (β = − 0.114, 95% CI − 0.180, − 0.049, P = 0.00064).

**Table 3:** Association between FAR levels and SIRI in different models.

	Model 1^a^ N = 3431 β (95% CI) P-value	Model 2^b^ N = 3431 β (95% CI) P-value	Model 3^c^ N = 3431 β (95% CI) P-value	Model 4^d^ N = 3431 β (95% CI) P-value
FAR per 1% decrease	− 0.141 (− 0.193, − 0.089) < 0.00001	− 0.140 (− 0.191, − 0.088) < 0.00001	− 0.125 (− 0.176, − 0.073) < 0.00001	− 0.114 (− 0.180, − 0.049) 0.00064
FAR quartile				
Q1 (< 5.5875%)	Reference	Reference	Reference	Reference
Q2 (5.5875–6.8018%)	− 1.212 (− 1.563, − 0.862) < 0.00001	− 1.206 (− 1.556, − 0.856) < 0.00001	− 1.040 (− 1.385, − 0.695) < 0.00001	− 1.037 (− 1.491, − 0.583) < 0.00001
Q3 (6.8018–8.4130%)	− 1.663 (− 2.014, − 1.312) < 0.00001	− 1.660 (− 2.010, − 1.309) < 0.00001	− 1.433 (− 1.780, − 1.086) < 0.00001	− 1.410 (− 1.855, − 0.964) < 0.00001
Q4 (6.4130–19.4871%)	− 1.318 (− 1.668, − 0.967) < 0.00001	− 1.308 (− 1.658, − 0.957) < 0.00001	− 1.148 (− 1.498, − 0.797) < 0.00001	− 1.117 (− 1.568, − 0.665) < 0.00001

^a^No adjustment.

^b^Adjusted for age, gender, BMI, ASA.

^c^Adjusted for age, gender, BMI, ASA, UA, Cr, UN, AST.

^d^Adjusted for age, gender, BMI, ASA, UA, Cr, UN, AST, PTH, calcium.

*FAR* fibrinogen-to-albumin ratio, *SIRI* systemic inflammation response index, *Q1* first quartile, *Q2* second quartile, *Q3* third quartile, *Q4* fourth quartile.

In comparison to Q1, SIRI in Model 4 decreased by 1.037, 1.410, and 1.117 in Q2, Q3, and Q4, respectively. In Q4 and Q3, OP patients showed a statistically significant decline in SIRI across all four models.

Subgroup analyses were conducted to ensure the robustness of Model 4. OPF patients were classified according to age, gender, BMI, ASA, UA, Cr, UN, AST, PTH, and calcium. The findings were then adjusted for the parameters that were not used in stratification. As shown in Table S1, all layers were stable, and a relatively consistent pattern was observed in these data.

### Analysis of thresholds and spline smoothing plots

**Figure 2:**
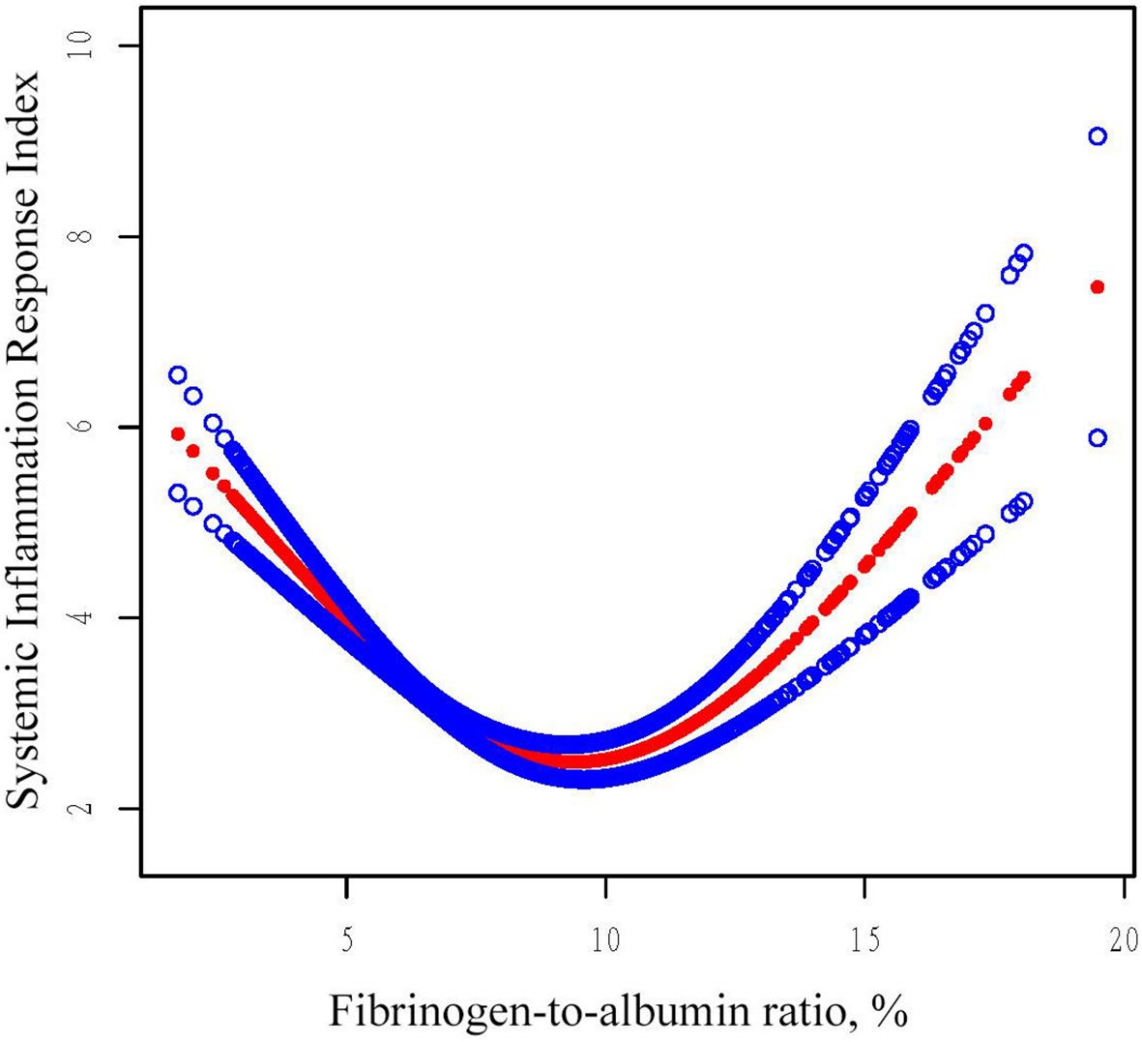
The relationship between FAR and SIRI. Adjusted smoothed curves corresponding to the relationship between FAR levels and SIRI. A generalized additive model revealed a thresholded non-linear relationship between FAR and SIRI in OPF patients. The upper and lower curves represent the range of the 95% confidence interval, and the middle curve represents the correlation between FAR and SIRI. Models were adjusted for age, gender, BMI, ASA, UA, Cr, UN, AST, PTH, and calcium. The red curve in Model 4 exhibited an inflection point (K) at 6.344%. *FAR* fibrinogen-to-albumin ratio, *SIRI* systemic inflammation response index, *BMI* body mass index, *ASA* American Society of Anesthesiologists, *UA* uric acid, *Cr* uric acid, *UN* uric acid, *AST* aspartate aminotransferase, *PTH* parathyroid hormone.

Figure 2 demonstrates the estimated exposure–response curve for FAR and SIRI in OPF patients stratified by BMI status. Once age, gender, ASA, UA, Cr, UN, AST, PTH, and calcium were adjusted for, GAM analysis showed a non-linear connection between FAR and SIRI in patients with BMI < 24 kg/m^2^, BMI between 24–28 kg/m^2^, and BMI > 28 kg/m^2^ (LRT < 0.001, LRT < 0.001, LRT = 0.006) (Table 4). Among the OPF patients included, there was a threshold non-linear connection between FAR and SIRI. Segmented linear regression models were utilized to ascertain the inflection point (K values were 6.667%, 5.151%, and 9.323%). The effect size, 95% confidence interval, and *P*-value for OPF patients with a BMI of less than 24 kg/m^2^ were, respectively, − 1.086, − 1.345, − 0.826, and < 0.0001. On the right side of the inflection point, the effect magnitude, 95% CI, and *P*-value were, in order, 0.130, 0.013, − 0.246, and 0.0294. The effect size, 95% confidence interval, and *P*-value for OPF patients with BMIs ranging from 24 to 28 kg/m^2^ were, respectively, − 2.141, − 2.932, − 1.350, and < 0.0001 on the left side of the inflection point. The effect size, 95% CI, and *P*-value for the positive side of the inflection point were 0.048, − 0.088, − 0.183, and 0.4934, respectively. These values suggest that there is no significant association. For OPF patients with a BMI greater than 28 kg/m^2^, there was no significant correlation observed on the left side of the inflection point. The effect size, 95% CI, and *P*-value were − 0.141, − 0.329, − 0.046, and 0.1411, respectively. The effect size, 95% confidence interval, and P-value on the right side of the inflection point were, in that order, 0.325, 0.105, − 0.544, and 0.0043.

**Table 4:** Threshold effect analysis examining the relationship between FAR and SIRI.

BMI	Model 4^a^			
	< 24 kg/m^2^ β (95% CI) *P*-value	24-28 kg/m^2^ β (95% CI) *P*-value	≥ 28 kg/m^2^ β (95% CI) *P*-value	Total
Model A^b^				*P*-interaction: 0.068
One line effect	− 0.167 (− 0.255, − 0.080) 0.0002	− 0.117 (− 0.239, 0.005) 0.0616	0.066 (− 0.046, 0.177) 0.2508	− 0.113 (− 0.179, − 0.048) 0.0007
Model B^c^				*P*-interaction: 0.081
FAR turning point (K), %	6.667	5.151	9.323	6.344
< K	− 1.086 (− 1.345, − 0.826) < 0.0001	− 2.141 (− 2.932, − 1.350) < 0.0001	− 0.141 (− 0.329, 0.046) 0.1411	− 1.062 (− 1.287, − 0.837) < 0.0001
> K	0.130 (0.013, 0.246) 0.0294	0.048 (− 0.088, 0.183) 0.4934	0.325 (0.105, 0.544) 0.0043	0.125 (0.041, 0.210) 0.0035
SIRI value at K	2.414 (2.093, 2.735)	2.967 (2.517, 3.417)	2.075 (1.479, 2.671)	2.522 (2.281, 2.764)
LRT test^d^	< 0.001	< 0.001	0.006	< 0.001

^a^Adjusted for age, gender, BMI, ASA, UA, Cr, UN, AST, PTH, calcium.

^b^Linear analysis, *P*-value < 0.05 indicates a linear relationship.

^c^Non-linear analysis.

^d^*P*-value < 0.05 means Model B is significantly different from Model A, which indicates a non-linear relationship.

*FAR* Fibrinogen-to-albumin ratio, *SIRI* systemic inflammation response index, *BMI* body mass index, *LRT* logarithmic likelihood ratio test.

## Discussion

Current research on inflammation and OP mostly focuses on the cellular level, uncovering several regulatory patterns and interactions between mediators of inflammation and osteoblasts and osteoclasts^18^. Using SIRI and FAR, two inflammatory markers, we performed a retrospective cross-sectional study in a group of OPF patients. Examining the correlation between FAR and SIRI in OPF patients was the aim of the study. For the first time, our results showed a non-linear relationship between FAR and SIRI in the OPF population of the Kunshan region of China. The study investigated the relationship between FAR and SIRI in detail using cross-sectional analysis and retrospective data from 3431 OPF patients. Following adjustments, the analysis showed that FAR and SIRI had a U-shaped association. A rise in FAR has a negative correlation with SIRI when it is less than 6.344%, with an impact size of − 1.062 (95% confidence interval − 1.287 to − 0.837, P < 0.0001). On the other hand, FAR exhibits a positive correlation with SIRI when it is more than 6.344%, with an effect size of 0.125 (95% confidence interval 0.041 to 0.210, P =0.0035). This discovery adds to our understanding of the mechanisms underlying the inflammatory response and offers a fresh viewpoint.

The few research that have examined the relationship between FAR levels and SIRI have not found one. The majority of prior research has looked at the prognostic implications and survival rates of various cancers concerning these two inflammatory markers independently; very few studies have examined the interaction between these two markers^19–21^. FAR is a blood biomarker used for prognostic and inflammatory assessment, as previous research has shown^22^. Many disease scenarios have been thoroughly examined and applied, such as COVID-19, cardiac issues, pancreatic cancer, hepatocellular carcinoma, bladder cancer, and newborn sepsis. The severity of the disease, patient survival rates, and prognostic indicators have all been connected to the FAR level^23^. The liver produces fibrinogen (FIB), a serum glycoprotein with a dimeric molecular structure that is essential to the physiology and pathology of inflammation and coagulation^24^. Fibrinogen production undergoes rapid upregulation during the acute phase of inflammation, encompassing bacterial infections, severe trauma, and surgical procedures^25^. Furthermore, there is a correlation between increased levels of plasma fibrinogen and persistent low-grade inflammation, platelet activation, greater production of adhesion molecules, stimulation of angiogenesis, and heightened infiltration of macrophages^26^. The liver produces albumin, which plays a significant role in both the acute inflammatory response and preserving plasma colloid osmotic pressure^27^. Nutritional and inflammatory circumstances affect its potential for synthesis^28^. Hypoalbuminemia is thought to be mostly caused by inflammation and malnutrition^29^. Elevated serum albumin levels inhibit the expression of vascular cell adhesion molecule-1, enhance the elimination of reactive oxygen species, and reduce inflammatory responses and endothelial cell death. The data suggest that albumin acts as an antioxidant and anti-inflammatory agent^30^. The physiological characteristics of serum albumin include anti-inflammatory, antioxidant, anticoagulant, antiplatelet aggregation, and capillary membrane stability maintenance^31^. SIRI represents the immune system's response to infection and invasive microorganisms^32^. This inflammatory index describes the immunological defense system, which comprises neutrophils, monocytes, and lymphocytes. SIRI serves as a prevalent prognostic and assessment indicator for several diseases. It has been extensively utilized in the management of acute pancreatitis, cardiovascular diseases, cancer, and stroke, among various other medical disorders. Physicians can assess a patient's inflammatory state and administer appropriate treatment based on anticipated clinical outcomes by monitoring alterations in the SIRI. Studies have demonstrated that SIRI is a separate predictor of outcome for patients with hepatoblastoma receiving neoadjuvant chemotherapy; the group with high SIRI had a significantly poorer 5-year overall survival than the group with low SIRI^33^. SIRI and other indicators are included in prognosis models for lower 5-year overall patients, which show great accuracy and reliability and allow prognostic risk assessment in these patients.

The liver responds to inflammation in its early stages by producing albumin, which has anti-inflammatory qualities and aids in the stability of capillary membranes^34^. Serum albumin levels consequently see a brief rise. Fibrinogen and serum albumin have a negative correlation^27^, and fibrinogen levels do not rise or slightly fall temporarily. As a result, FAR is decreased in the initial stages of inflammation. Lymphocytes migrate to the site of inflammation from the bloodstream as a result of the release of inflammatory cytokines and the actions of inflammatory mediators. Consequently, lymphocytes tend to decrease in number during the initial phases of inflammation^35^. This migratory process may cause the blood's lymphocyte numbers to momentarily drop, which would cause a brief rise in SIRI. Therefore, when FAR is less than 6.344% in the early phases of inflammation, there is a negative association between FAR and SIRI. Serum albumin levels dramatically drop in the ultimate stage of inflammation. This results in decreased albumin synthesis or increased loss and is caused by prolonged inflammatory activation and metabolic alterations^36^. Moreover, inflammation may enhance vascular permeability, which may facilitate albumin loss and leakage^37^. As a result, the level of plasma albumin may decrease even further during advanced stages of inflammation. Hypoalbuminemia in late-stage inflammation leads to an increase in the production of lipoproteins and procoagulant factors such as factor V, factor VIII, and fibrinogen as a compensatory reaction, due to the negative association between serum albumin and fibrinogen. As a result, hyperlipidemia occurs, leading to an increased risk of blood clotting due to considerably raised levels of fibrinogen^27,38^. FAR is hence raised in late-stage inflammatory conditions. White blood cell counts, especially those of neutrophils, usually rise in the latter stages of inflammation^39^. As a component of the immune system, WBCs are primarily responsible for recognizing and getting rid of infections, cleaning up injured tissue, and controlling the inflammatory response^40^. When inflammation arises, the bloodstream carries WBCs to the inflammatory site, where they contribute to tissue repair and the regulation of the inflammatory response^41^. WBCs are drawn to the area of inflammation by the release of inflammatory mediators including chemokines and cytokines, which cause the circulation to diverge^42^. White blood cells penetrate the inflammatory region by adhering to endothelial cells and navigating the artery wall^43^. In response to inflammatory stimuli, hematopoietic stem cells and precursor cells in the bone marrow can proliferate and develop to produce more WBC, including neutrophils, lymphocytes, monocytes, etc.^44^. SIRI significantly rises as a result of this. Consequently, FAR is positively connected with SIRI in the late stage of inflammation, when FAR is greater than 6.344%. Systemic inflammation and fibrinogen have been found to positively correlate in previous research, whereas albumin and systemic inflammation have been found to negatively correlate^45^. As a result, while FAR is strongly associated with systemic inflammation and SIRI measures the extent of systemic inflammation, additional evidence supports the direct correlation between FAR and SIRI in advanced stages of inflammation.

One of the main factors contributing to the development of OP is inflammation. Serum levels of IL-1β, IL-6, and F-I type collagen N-terminal peptide (NTx) were found to be considerably higher in postmenopausal women with OP compared to the control group in a study conducted by Al-Daghri and colleagues^46^. These investigations have shown that inflammation, which is controlled by patterns of cytokines, plays a crucial role in the development and progression of OP. Persistent systemic inflammation increases the probability of acquiring many diseases, such as periodontal and cardiovascular conditions. Chronic inflammation has been linked to several elements essential to bone physiology and may be a risk factor for OP, according to a study by several scientists^47^. Important inflammatory mediators such as IL-1, IL-6, and tumor necrosis factor-alpha (TNF-α) are secreted by a variety of cells, including neutrophils and macrophages. The primary factors that activate osteoclasts are TNF-α and IL-1, although IL-6 works in concert with other factors that promote bone^48^. Furthermore, a negative connection was noted by Ganesan et al. between bone mineral density (BMD) and high-sensitivity C-reactive protein (hs-CRP), which is produced by IL-6^49^. In addition, they proposed a correlation between OP and inflammation. Additionally, hs-CRP levels and BMD were reported to be negatively correlated by Koh et al.^49^. The expanding body of evidence that supports the association between inflammation and OP is bolstered by these findings. Consequently, by employing two novel inflammation indicators, FAR and SIRI, we are capable of assessing the levels of systemic inflammation within a specific population and implementing timely measures to mitigate the likelihood of OP. The body is in the early stages of inflammation and needs prompt intervention to lower inflammation levels and avoid inflammatory OP when FAR is less than 6.344% and SIRI is higher.

This is the first study of its kind in China to investigate the independent correlation between the SIRI and FAR of an OPF individual. Our research might have immediate implications for clinical practice. One potential implication of the observed nonlinear correlation between FAR and SIRI is that elevated FAR levels should be considered in the assessment of inflammatory markers, in addition to elevated SIRI levels. Assessing the progression of inflammation is dependent upon this critical marker. Early-stage inflammation is suggested when SIRI is high and FAR is less than 6.344%. Inflammation occurs either in the chronic or terminal state when FAR is more than 6.344% and SIRI is high. Considering these conditions, we propose the concept of an "inflammatory trough," which denotes the stage at which patients with OPF are expected to encounter the least amount of inflammation, as indicated by a FAR of 6.344%. Understanding this particular facet has significant implications for the medical diagnosis and treatment of patients with OPF. These markers could also be included in the clinical evaluation of patients' OPF risk prediction^50^. Moreover, this finding provides direction for clinical interventions that attempt to mitigate inflammation in patients with osteoporosis. Additionally, it can aid in the development of clinical procedures and treatment protocols that are appropriate for specific OP patients.

This analysis contains several limitations. Firstly, as this was a retrospective cross-sectional study, there is no evidence of a causal relationship between FAR and SIRI. Furthermore, even though several covariates were considered, only the association between FAR and SIRI was investigated, meaning that residual confounding variables such as the effects of medication cannot be completely excluded from the research. Further planned and stratified cohort studies, appropriate control groups, and confounding factor accounting are needed to further understand the relationship between FAR and SIRI. Third, because of the single-center design and very small sample size, it was not possible to extrapolate the findings to other ethnic groups. This study highlights the need for more research using big studies that involve people of varied ethnicities, multi-center RCTs, and other biochemical indicators to better assure the reliability of these study results.

## Conclusion

In summary, the results of our investigation indicate that there is a U-shaped relationship between FAR and SIRI in people with OPFs. When FAR is less than 6.344%, there is a negative correlation between FAR and SIRI. Conversely, when FAR exceeds 6.344%, there is a positive correlation between FAR and SIRI. Thus, in the context of diagnosing and treating OP patients, we propose the concept of an "inflammatory trough," which is the lowest inflammatory state that is likely to be observed in OPF patients with a FAR of 6.344%. The findings enable the early identification of people who may be at risk for OP and allow early preventive actions to be implemented to lower the risk of OP. However, additional follow-up studies involving a larger patient population would be required to validate these findings.

### Supplementary Information

Supplementary Table S1.

Supplementary Information 2.

## Data Availability

Data is provided within the manuscript or supplementary information files.
