# Supplementary material for: U-shaped relationship found between fibrinogen-to-albumin ratio and systemic inflammation response index in osteoporotic fracture patients
Source: Sci Rep. 2024 May 17;14:11299. doi: 10.1038/s41598-024-61965-9 (PMC11101643; doi:10.1038/s41598-024-61965-9)
Supplement: Supplementary file 1 — Supplementary Table S1. [file 41598_2024_61965_MOESM1_ESM.docx]

Table S1. Subgroup Analyses Exploring the Association Between FAR and SIRI

|  | N | β (95% CI) *P*-value | |
| --- | --- | --- | --- |
| Age tertile, y |  | |  |
| Low | 1136 | | -0.1340 (-0.2460, -0.0221) 0.0192 |
| Middle | 1085 | | -0.0806 (-0.2021, 0.0410) 0.1943 |
| High | 1210 | | -0.1654 (-0.2764, -0.0545) 0.0036 |
| Gender, N |  | |  |
| Female | 2334 | | -0.1204 (-0.1980, -0.0427) 0.0024 |
| Male | 1097 | | -0.1306 (-0.2523, -0.0088) 0.0359 |
| BMI tertile, kg/m^2^ (Chinese criterion) |  | |  |
| <24 | 2114 | | -0.1671 (-0.2546, -0.0797) 0.0002 |
| 24-28 | 1079 | | -0.1169 (-0.2393, 0.0055) 0.0616 |
| ≥28  BMI tertile, kg/m^2^ (WHO criterion)  <25  25-30  ≥30 | 238  2525  826  80 | | 0.0657 (-0.0460, 0.1774) 0.2508  -0.1359(-0.2124, -0.0595) 0.0005  -0.0839(-0.2175, -0.0498) 0.2189  -0.0906(-0.2399, 0.4212) 0.5910 |
| ASA tertile, N |  | |  |
| 1 | 311 | | -0.1112 (-0.3339, 0.1114) 0.3288 |
| 2 | 2308 | | -0.1003 (-0.1843, -0.0162) 0.0195 |
| 3 | 801 | | -0.1566 (-0.2708, -0.0424) 0.0074 |
| 4 | 11 | | NA |
| UA tertile, μmol/L |  | |  |
| 61 - 239 | 1143 | | -0.0782 (-0.1711, 0.0147) 0.0994 |
| 240 - 311 | 1135 | | -0.1891 (-0.3108, -0.0673) 0.0024 |
| 312 - 997 | 1153 | | -0.1215 (-0.2521, 0.0091) 0.0687 |
| Cr tertile, μmol/L |  | |  |
| 21 - 54 | 1086 | | -0.1458 (-0.2596, -0.0320) 0.0123 |
| 55 - 68 | 1197 | | -0.2874 (-0.4167, -0.1581) <0.0001 |
| 69 - 982 | 1148 | | 0.0321 (-0.0630, 0.1272) 0.5082 |
| UN tertile, mmol/L |  | |  |
| 1.5 - 4.89 | 1089 | | -0.2039 (-0.3089, -0.0990) 0.0002 |
| 4.9 - 6.35 | 1198 | | -0.2032 (-0.3173, -0.0892) 0.0005 |
| 6.36 - 139.81 | 1144 | | -0.0395 (-0.1577, 0.0787) 0.5124 |
| AST tertile, U/L |  | |  |
| 8 - 19 | 1130 | | -0.0812 (-0.1731, 0.0107) 0.0837 |
| 20 - 25 | 1065 | | -0.1104 (-0.2110, -0.0098) 0.0318 |
| 26 - 822 | 1236 | | -0.0950 (-0.2523, 0.0623) 0.2369 |
| PTH tertile, pmol/L |  | |  |
| 0 - 10.4 | 712 | | -0.1725 (-0.2779, -0.0672) 0.0014 |
| 10.41 - 14.87 | 719 | | -0.1543 (-0.2673, -0.0413) 0.0076 |
| 14.88 - 213.99 | 721 | | -0.0672 (-0.1877, 0.0533) 0.2747 |
| calcium tertile, mmol/L |  | |  |
| 1.09 - 2.15 | 1113 | | -0.1168 (-0.2368, 0.0031) 0.0567 |
| 2.16 - 2.25 | 1127 | | -0.0396 (-0.1404, 0.0611) 0.4409 |
| 2.26 - 2.91 | 1189 | | -0.2466 (-0.3627, -0.1306) <0.0001 |

Adjusted for age, gender, BMI, ASA, UA, Cr, UN, AST, PTH, calcium.

Abbreviations: BMI, body mass index; ASA, American Society of Anesthesiologists; UA, uric acid; Cr, creatinine; UN, urea nitrogen; AST, aspartate aminotransferase; PTH, parathyroid hormone.
